# Low KIF26B Expression Reduces Paclitaxel Resistance and Predicts Good Prognosis in Ovarian Cancer

**DOI:** 10.3390/cimb48020226

**Published:** 2026-02-20

**Authors:** Yuting Su, Xia Liu, Yue Yu, Xiaoying Chen, Lizhou Shi, Zhe Du, Yuang Mao, Fuqiang Yin

**Affiliations:** 1 Collaborative Innovation Centre of Regenerative Medicine and Medical BioResource Development and Application Co-Constructed by the Province and Ministry, Guangxi Medical University, Nanning 530021, China; 2 Life Sciences Institute, Guangxi Medical University, Nanning 530021, China; 3 Key Laboratory of Longevity and Aging-Related Diseases of Chinese Ministry of Education, Institute of Neuroscience and Guangxi Key Laboratory of Brain Science, School of Basic Medical Sciences, Guangxi Medical University, Nanning 530021, China; 4 Guangxi Health Commission Key Laboratory of Basic Research on Brain Function and Disease, Nanning 530021, China; 5 Key Laboratory of Human Development and Disease Research (Guangxi Medical University), Education Department of Guangxi Zhuang Autonomous Region, Nanning 530021, China; 6 Pharmaceutical College, Guangxi Medical University, Nanning 530021, China; 7 Key Laboratory of High-Incidence-Tumor Prevention and Treatment (Guangxi Medical University), Ministry of Education, Nanning 530021, China

**Keywords:** KIF26B, ovarian cancer, drug resistance, paclitaxel, SLC7A11

## Abstract

Ovarian cancer, the most lethal type of tumour of the female reproductive system, severely threatens women’s life and health. Despite paclitaxel being a key chemotherapeutic agent in the standard treatment for ovarian cancer, the majority of patients eventually develop resistance to paclitaxel, constituting a significant obstacle to successful treatment. KIF26B, a kinesin family protein, is involved in various cancers, but its role in ovarian cancer and chemotherapy resistance is unclear. In this study, we evaluated the role of KIF26B in drug-resistant ovarian cancer and the underlying mechanisms. Bioinformatics analysis revealed that KIF26B was highly expressed in ovarian cancer tissues and was associated with poor clinical characteristics. Moreover, KIF26B expression was consistently high in chemotherapy-resistant tissues across multiple treatment subgroups, with ROC curve analyses confirming its predictive power for chemoresistance, particularly in advanced serous ovarian cancer. To further investigate the role of KIF26B in ovarian cancer resistance, the effects of KIF26B on cell proliferation, colony formation, the cell cycle, apoptosis, and microtubule polymerization under paclitaxel treatment were assessed. KIF26B knockdown significantly reduced paclitaxel resistance in ovarian cancer cells, inhibited cell proliferation, and promoted apoptosis. Furthermore, KIF26B interference induced cell cycle arrest and altered microtubule polymerization dynamics in paclitaxel-resistant cells. Additionally, our analyses revealed a negative correlation between KIF26B and SLC7A11 in ovarian cancer, particularly in chemoresistant tissues. Combined KIF26B and SLC7A11 expression provided stronger prognostic value than either gene alone did, and functional assays demonstrated that SLC7A11 contributed to the regulation of the KIF26B-mediated paclitaxel response. Overall, our results indicate that KIF26B is crucial for ovarian cancer progression and chemotherapy resistance, likely through SLC7A11 regulation. KIF26B may serve as a potential therapeutic target for overcoming paclitaxel resistance.

## 1. Introduction

Ovarian cancer, recognized as the leading cause of death among gynaecological malignancies, is often referred to as the “silent killer” of women because of its high mortality rate and insidious onset [1]. According to the 2020 GLOBOCAN report, there were 313,959 newly diagnosed ovarian cancer cases worldwide, ranking it the 7th most common malignancy among women, with 207,252 related deaths [2]. Ovarian cancer severity is largely attributed to its lack of early symptoms and the absence of effective preventive and screening methods. These factors underscore the critical need for further research and timely intervention. Over the past two decades, advances in surgical techniques and the standardized use of taxane- and carboplatin-based chemotherapy, as well as maintenance therapies, have significantly improved patient outcomes. However, the 5-year survival rate for advanced-stage patients remains below 30%, and approximately 70% of patients experience relapse because of the development of chemotherapy resistance following initial treatment [3]. This current situation highlights the need to understand the molecular mechanisms driving tumour progression and chemotherapy resistance to improve patient prognosis.

Kinesin superfamily proteins (KIFs) are a class of molecular motor proteins consisting of 45 known members and are divided into 14 subfamilies [4]. Different members of the kinesin superfamily play diverse roles within cells, including functions in mitosis (i.e., cell division) and intracellular vesicle and organelle transport [5]. KIFs perform various essential biological functions and are indispensable for cellular activities. Numerous studies have demonstrated that KIFs play crucial roles in the initiation and progression of various cancers, with alterations in their expression levels closely associated with the occurrence and development of several malignancies. Therefore, in-depth research on KIFs, particularly strategies targeting KIFs in combination with chemotherapy, may provide new therapeutic targets for cancer treatment [6]; this is not only critical for advancing cancer therapy but also offers novel strategies and insights for clinical treatment. KIF26B, a member of the kinesin-11 family, is involved primarily in intracellular transport and has been shown to play a role in processes such as mitosis, migration, and cellular organization. While several KIF proteins have been linked to cancer progression, the role of KIF26B in ovarian cancer has been less extensively studied. Recent research has indicated that KIF26B is involved in regulating key pathways related to cell proliferation and survival. For instance, high KIF26B expression is directly associated with poor prognosis in colorectal and breast cancers [7,8]. Additionally, KIF26B has been linked to non-small cell lung cancer [9], suggesting its potential involvement in tumorigenesis. KIF26B is involved in tumour development, an increased risk of metastasis, poor prognosis, and the development of drug resistance; however, its specific role in ovarian cancer, particularly in the context of chemotherapy resistance, remains unclear.

**Figure 1:**
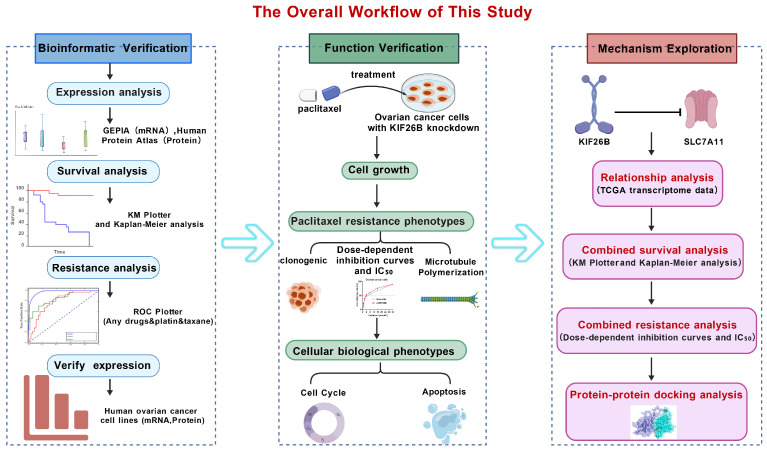
Schematic overview of the study design investigating the role of KIF26B in paclitaxel resistance and prognosis of ovarian cancer.

Given the urgent need to develop new therapeutic targets for ovarian cancer and associated ovarian cancer drug resistance, in this study, we systematically analysed KIF26B expression in ovarian cancer and its relationship with prognosis and drug resistance. Special emphasis was placed on determining the role of KIF26B in disease progression, chemotherapy resistance, and prognosis assessment in ovarian cancer. Unveiling the molecular functions of KIF26B may provide novel insights for developing targeted therapies, thereby improving treatment efficacy and reducing chemotherapy resistance in ovarian cancer patients. The overall workflow of this study is shown in Figure 1.

## 2. Materials and Methods

### 2.1. Data Acquisition

Expression data of *KIF26B* in ovarian cancer were obtained from Gene Expression Profling Interactive Analysis (GEPIA, http://gepia.cancer-pku.cn, accessed on 9 February 2026), integrating The Cancer Genome Atlas (TCGA) normal and Genotype Tissue Expression (GTEx) data. Differential expression between ovarian cancer tissues (*n* = 426) and normal ovarian tissues (*n* = 88) was analyzed using log2(TPM + 1) values, with significance assessed by unpaired t-test (|log2FC| ≥ 1, *p* < 0.01). The expression levels of *KIF26B* in different clinical stages were evaluated through The University of Alabama at Birmingham CANcer data analysis Portal (UALCAN, http://ualcan.path.uab.edu, accessed on 9 February 2026) based on TCGA ovarian cancer cohort, including 20 stage II, 243 stage III, and 38 stage IV tissues. Protein-level validation was performed using immunohistochemistry (IHC) data from the Human Protein Atlas (https://www.proteinatlas.org/, accessed on 9 February 2026, antibodies HPA028478, HPA028561, HPA028562, and HPA027709), with staining categorized as not detected, low, medium, or high. Chemotherapy response data from 1347 ovarian cancer patients were analyzed using the ROC Plotter database (https://rocplot.org/, accessed on 9 February 2026), with resistance defined as relapse within six months following treatment. The association between *KIF26B* expression and chemotherapy resistance was assessed across different treatment regimens (any, taxane, or platinum) and further stratified by histology (serous), tumor grade (III), and stage (III).

### 2.2. Cell Culture

The human ovarian cancer cell line HeyA8 (H) was kindly provided by Professor Fengxia Xue from Tianjin Medical University [10]. Paclitaxel-resistant ovarian cancer cell line HeyA8-R (H-R) was generated by stepwise increased concentrations of paclitaxel, with the concentration ranging from 2 nmol/L to 100 nmol/L, over 13 months. The H-R cells as described above were incubated with gradual increasing concentrations of paclitaxel for 24 h, and then cultured in paclitaxel-free medium until cells grew well. Thereafter, the cells were incubated with gradual increasing concentrations of paclitaxel for a further 24 h [11]. Both H and H-R cells were cultured in RPMI-1640 medium (Wisent, Nanjing, China) supplemented with 10% foetal bovine serum at 37 °C and 5% CO_2_. The resistance index (RI), defined as the ratio of the half-maximal inhibitory concentration (IC_50_) value of paclitaxel in paclitaxel-resistant H-R cells to that in parental cells, was 5.42 ± 0.55.

### 2.3. Real-Time Quantitative Polymerase Chain Reaction

Total RNA was isolated from cultured cells using TRIzol reagent (Thermo Fisher Scientific, Waltham, MA, USA) and quantified with NanoDrop 2000 spectrophotometer. First-strand cDNA was synthesized using a PrimeScript™ RT reagent Kit with gDNA Eraser (Takara, Shiga, Japan, #RR047A). Real-time quantitative polymerase chain reaction (RT-qPCR) analysis was conducted using ChamQ Universal SYBR qPCR Master Mix (Vazyme, Nanjing, China, #P525). All the RT-qPCR assays were performed on an ABI Prism 7300 system (Applied Biosystems, Foster City, CA, USA). After the reactions were complete, the relative expression levels of the samples were calculated using the 2^−∆∆Ct^ method, with *GAPDH* used as the internal reference. The primer sequences for *KIF26B* were 5′-CTGAACTCGGTAAATGGGAACC-3′ and 5′-GTAGTTTAGCCTGTTCAGCCAG-3′. The primer sequences for GAPDH were 5′-CAGCCTCAAGATCATCAGCAAT-3′ and 5′-AGTCCTTCCACGATACCAAAGT-3′.

### 2.4. Lentivirus Transfection

To create stable cell lines with KIF26B knockdown, lentiviral particles targeting the KIF26B gene provided by Cyagen (Santa Clara, CA, USA) were used. Target cells, including H and H-R cells, were seeded in 24-well plates and infected when the cell density reached approximately 50–70%. The lentiviral particles were added to the culture medium at a multiplicity of infection (MOI) of 80 to ensure efficient infection. After 12–16 h, the virus-containing medium was replaced with fresh complete medium, and the cells were cultured for an additional 48 h. The cells were subsequently selected with 1 µg/mL puromycin-containing medium for 1–2 weeks until the uninfected cells died and successfully infected cells proliferated. The stable cell populations were expanded, and the efficiency of KIF26B knockdown was confirmed via RT-qPCR and Western blotting; both the mRNA and protein levels were measured to ensure a significant reduction in KIF26B expression.

### 2.5. Western Blotting

Total protein was extracted from cells using RIPA buffer supplemented with protease and phosphatase inhibitors. Protein concentrations were determined via the BCA assay, and equal amounts of protein (20–50 µg) were separated by SDS-PAGE and transferred to PVDF membranes. After blocking with 5% nonfat milk in TBST, the membranes were incubated overnight at 4 °C with an anti-KIF26B antibody (1:750 dilution, Proteintech, Rosemont, IL, USA, #17422-1-AP). Following washes, the membranes were incubated with an HRP-conjugated secondary antibody (1:10,000) for 1 h at room temperature. Protein bands were visualized using enhanced chemiluminescence (ECL) reagents and detected with a gel imaging system. Semiquantitative analysis was performed with ImageJ 1.x software.

### 2.6. CCK-8 Assay

Cell growth curves were assessed with CCK-8 assays (APExBIO Technology LLC, Houston, TX, USA, # K1018). Stable KIF26B-knockdown and matched control cells from the H and paclitaxel-resistant H-R lines were seeded in 96-well plates. For growth curves, 200–400 cells/well were cultured and assessed at 24, 48, 72, 96, 120, and 144 h; 10 μL of CCK-8 was added to 100 μL of medium per well; the cells were incubated at 37 °C for 1–2 h. Absorbance at 450 nm was measured using a microplate reader, and growth curves were generated based on the relative optical density values.

### 2.7. CellTiter-Glo^®^Luminescent Cell Viability Assay

The viability of H-shKIF26B, H-Scramble, H-R-shKIF26B, and H-R-Scramble cells was quantified using the CellTiter-Glo^®^ Luminescent Cell Viability Assay (Beyotime, Shanghai, China, #C0065S). Cells were seeded in white, opaque 96-well plates at 1200 cells/well and allowed to adhere for 16–18 h. Paclitaxel was then applied at 31, 16, 8, 4, 2 and 1 nmol/L (H-Scramble/H-shKIF26B cells) or 125, 63, 31, 16, 8, and 4 nmol/L (H-R-Scramble/H-R-shKIF26B cells) in triplicate, followed by incubation for 72 h. The cells were equilibrated to room temperature for 10 min, CellTiter-Glo^®^ reagent was added in accordance with the instructions of the kit, the plates were gently shaken for 2 min, and luminescence was recorded after 10 min of stabilization in luminescence mode. Background signals from blank wells were subtracted, the viability was normalized to that of the vehicle control, dose–response curves were fit by nonlinear regression (variable-slope, four-parameter logistic), and the IC_50_ values were calculated and compared between the knockdown and control groups within each cell background.

### 2.8. Colony Formation Assay

Stable KIF26B-knockdown H and H-R cell lines (H-shKIF26B and H-R-shKIF26B cells) and their negative controls were seeded in 6 cm dishes (600 cells/dish) and allowed to adhere for 16–18 h. Paclitaxel was then applied at 2 or 4 nmol/L (H-Scramble/H-shKIF26B cells) and 16 or 32 nmol/L (H-R-Scramble/H-R-shKIF26B cells) in triplicate. The cultures were maintained for 9 days, and drug-containing medium was replaced every 2–3 days. Colonies were washed with DPBS, fixed with 4% paraformaldehyde (25 min, RT), stained with 0.1% crystal violet (30 min), rinsed, air-dried, and imaged by inverted microscopy. Colony formation was quantified using ImageJ, and the rates were compared across paclitaxel doses to evaluate the effect of KIF26B knockdown on drug resistance.

### 2.9. Microtubulin (α-Tubulin) Immunofluorescence Staining Assay

H and H-R KIF26B-knockdown cells (H-shKIF26B and H-R-shKIF26B cells) and control cells (H-Scramble and H-R-Scramble cells) were seeded on coverslips, grown to 50–70% confluence, and treated with paclitaxel for 72 h (*n* = 3). The cells were fixed (4% paraformaldehyde, 20 min, RT), washed with DPBS (3 × 5 min), permeabilized (0.1% Triton X-100/PBS, 30 min), and blocked (5% BSA/PBS, 1 h). The cells were incubated with a primary anti-α-tubulin antibody (1:300) overnight at 4 °C, followed by incubation with an Alexa Fluor 555–conjugated secondary antibody (1:500, 1 h, RT, protected from light). Nuclei were counterstained with DAPI, coverslips were mounted, and images were acquired using an epifluorescence microscope under identical settings. Red fluorescence intensity was quantified to assess microtubule polymerization.

### 2.10. Cell Cycle and Apoptosis Analysis

H-shKIF26B, H-Scramble, H-R-shKIF26B, and H-R-Scramble cells were seeded in 6 cm culture dishes and treated with various concentrations of paclitaxel for 72 h, with three replicates per group. Upon reaching 70–80% confluence, the cells were washed with DPBS, collected via trypsin digestion, and stained for cell cycle analysis with DNA staining and permeabilization solution (Lianke Biotechnology Co., Ltd., Hangzhou, China, #CCS012) or for apoptosis analysis with Annexin V-FITC/7-AAD (Lianke Biotechnology Co., Ltd., Hangzhou, China, #AP104). After a 30 min incubation in the dark, the cells were analysed by flow cytometry (Beckman Coulter, Inc., Brea, CA, USA). For cell cycle analysis, propidium iodide (PI) was used to assess distribution across the G_0_/G_1_, S, and G_2_/M phases. For apoptosis analysis, the cells were categorized as early apoptotic (Annexin V-FITC positive/7-AAD negative), late apoptotic (Annexin V-FITC positive/7-AAD positive), or dead cells (Annexin V-FITC negative/7-AAD positive) cells. A minimum of 10,000 events were analysed per sample. ModFit LT 4.x software was used to process the cell cycle data, and flow cytometry software was used to determine the apoptosis rates. The impact of KIF26B knockdown on paclitaxel resistance was assessed by comparing the cell cycle distribution and apoptosis rates between knockdown and control cells at different paclitaxel concentrations.

### 2.11. Molecular Docking Analysis

Protein-protein docking between KIF26B and SLC7A11 was carried out using the HDOCK server. The amino acid sequences of KIF26B and SLC7A11 were obtained from the UniProt database (https://www.uniprot.org, accessed on 9 February 2026) and submitted to HDOCK (http://hdock.phys.hust.edu.cn/, accessed on 9 February 2026) in FASTA format for sequence-based docking. All parameters were kept at their default settings. The top-ranked docking model was selected for subsequent analysis. Interfacial interactions were analyzed using LigPlot+ version 2.2.4 to identify hydrogen bonds, salt bridges, and hydrophobic contacts. In LigPlot+ analysis, KIF26B and SLC7A11 were assigned as chain A and chain B, respectively. Visualization of the docking conformation was performed using PyMol version 2.2.0. KIF26B was displayed using a cartoon representation colored in blue-purple, whereas SLC7A11 was shown in stick representation colored in cyan. Residues located at the predicted binding interface were highlighted accordingly. Docking scores were obtained from the HDOCK output files.

### 2.12. Correlation and Survival Analyses

*KIF26B* and *SLC7A11* mRNA expression in ovarian cancer was analysed using TCGA data (*n* = 489). The expression values were standardized and log2 transformed. Pearson correlations were computed for the full cohort and for the chemotherapy-sensitive (*n* = 197) and chemotherapy-resistant (*n* = 90) subsets; two-sided *p* < 0.05 was considered significant. Survival associations were evaluated in the Kaplan-Meier plotter (https://kmplot.com/analysis/), accessed on 9 February 2026, ovarian cohort, which comprised a total of 1815 patients, with 1656 patients overall survival (OS) data, 1435 patients progression-free survival (PFS) data, and 782 patients post-progression survival (PPS) data. Patients were dichotomized into high vs. low expression using the platform’s autoselected cut-offs for KIF26B and SLC7A11. Combined prognostic effects were assessed by cross-classifying KIF26B and SLC7A11 expression into four groups (L + H, H + H, L + L, and H + L).

### 2.13. Statistical Analysis

During the data analysis phase, we used SPSS 26.0 and GraphPad Prism 9.5 for data analysis. For continuous data conforming to a normal distribution, we employed descriptive statistics in the form of the mean ± standard deviation. For comparisons between the means of two groups, the t test was applied, whereas for comparisons involving multiple groups, we opted for analysis of variance (ANOVA). Additionally, we set *p* < 0.05 as the significance level for significant differences, ensuring the reliability and validity of our research findings. This rigorous data analysis approach provides solid statistical support for the conclusions drawn in this study.

## 3. Results

### 3.1. High KIF26B Expression Is Associated with Poor Prognosis and Chemotherapy Resistance in Ovarian Cancer

**Figure 2:**
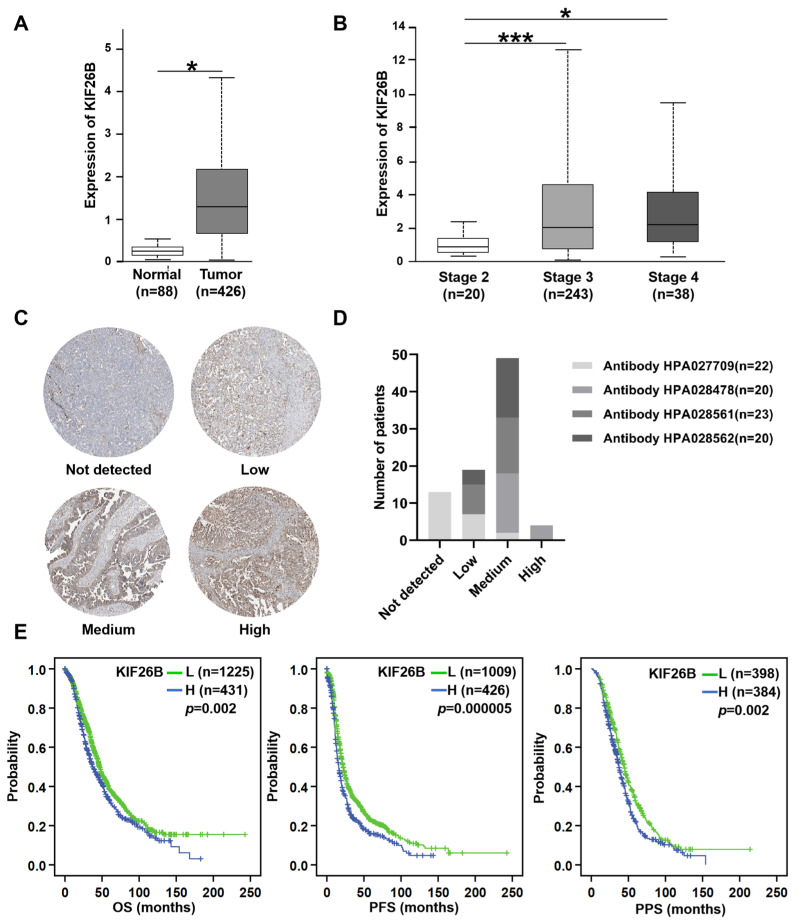
KIF26B expression in ovarian cancer and its correlation with clinical stage and prognosis. (**A**) *KIF26B* mRNA expression levels in 88 normal ovarian tissues (Normal) and 426 ovarian cancer tissues (Tumor) based on RNA-seq data from TCGA and GTEx datasets. (* *p* < 0.05). (**B**) *KIF26B* mRNA expression levels in 20 stage II ovarian cancer tissues (Stage 2), 243 stage III ovarian cancer tissues (Stage 3), and 38 stage IV ovarian cancer tissues (Stage 4) derived from TCGA ovarian cancer cohort. (* *p* < 0.05, *** *p*< 0.001). (**C**,**D**) Protein expression levels of KIF26B in ovarian cancer tissues were assessed using four independent antibodies (HPA027709, HPA028478, HPA028561, HPA028562) from the Human Protein Atlas database. The representative IHC images of four expression levels (not detected, low, medium, high) are displayed alongside a column graph quantifying the number of patients at each expression level per antibody. (**E**) Kaplan-Meier analysis was performed to evaluate the association of KIF26B expression with OS, PFS and PPS in patients with ovarian cancer. The KIF26B level was classified as low (L) and high (H) using the auto-selected best cut-off value. OS, overall survival; PFS, progression-free survival; PPS, postprogression survival.

To explore the potential role of KIF26B in ovarian cancer progression and treatment response, we first analyzed its expression levels in 426 ovarian cancer tissues compared to 88 normal ovarian tissues. *KIF26B* mRNA expression was significantly higher in ovarian cancer tissues than in normal ovarian tissues. (*p* < 0.05, Figure 2A). Further analysis was conducted to assess KIF26B expression across different ovarian cancer stages. For stage II (*n* = 20), stage III (*n* = 243), and stage IV (*n* = 38) ovarian cancer tissues, *KIF26B* expression was significantly higher in stage III tissues than in stage II tissues (*p* < 0.001). Similarly, in stage IV tissues, KIF26B expression was significantly augmented compared to stage II tissues (*p* < 0.05, Figure 2B).

KIF26B protein expression levels were assessed using IHC staining data retrieved from the Human Protein Atlas database. While antibody HPA027709 (*n* = 22) primarily showed unstained or low expression, substantial expression was consistently detected with the other three antibodies. Specifically, HPA028478 (*n* = 20) revealed exclusively medium (80%) or high (20%) expression of KIF26B protein. Similarly, HPA028561 (*n* = 23) and HPA028562 (*n* = 20) showed predominantly medium expression of KIF26B protein (65.2% and 80%, respectively), with the remaining cases being low expression. The representative images of KIF26B IHC and the corresponding expression profile are shown in Figure 2C,D. These findings corroborated the mRNA expression data, indicating that KIF26B is expressed at varying levels in ovarian cancer tissues.

Kaplan–Meier survival analysis was conducted to assess the correlation between KIF26B expression and survival outcomes in a cohort of 1815 ovarian cancer patients comprising 1656 with OS data, 1435 with PFS data, and 782 with PPS data. It revealed that high KIF26B expression was significantly associated with poor OS, PFS, and PPS (*p* = 0.002, *p* = 0.000005, and *p* = 0.002, respectively), as shown in Figure 2E. The log-rank test was used for statistical analysis, and the results confirmed the prognostic significance of KIF26B expression in ovarian cancer. These findings suggest that high KIF26B expression is a strong predictor of poor prognosis and may serve as a potential biomarker for patient stratification and targeted therapies.

**Figure 3:**
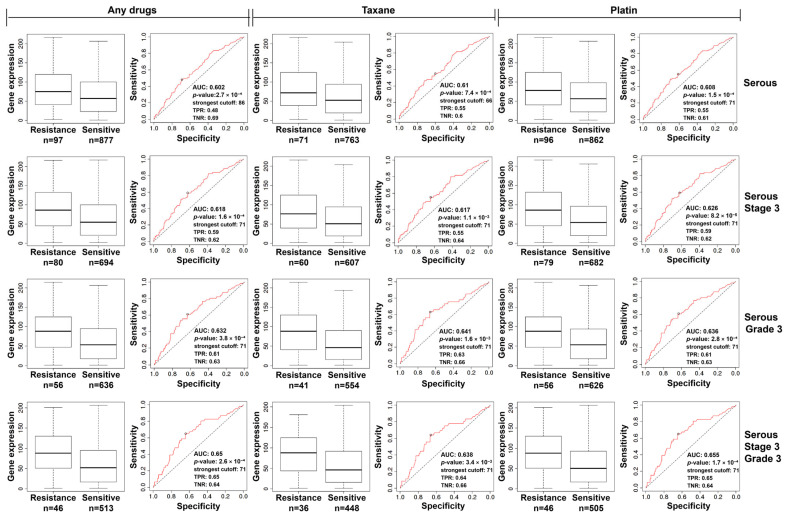
*KIF26B* expression in chemoresistant ovarian cancer tissues and the prediction of therapeutic response. Using a large sample of 1347 ovarian cancer cases, ROC plotter was employed to analyse *KIF26B* expression in chemoresistant (Resistance) and chemosensitive (Sensitive) tissues, as well as its correlation with chemotherapy resistance prediction in ovarian cancer. “Any drugs” refers to all chemotherapy drugs; “Taxane” refers to paclitaxel; “Platin” refers to platinum-based drugs; “Serous” refers to serous ovarian cancer; “Stage 3” refers to stage III ovarian cancer; and “Grade 3” refers to poorly differentiated grade III ovarian cancer. “Taxane-serous-Stage 3-Grade 3” indicates tissues from patients with poorly differentiated stage III serous ovarian cancer treated with paclitaxel, and a similar classification applies to other patients. The classification of chemoresistant and chemosensitive tissues is based on whether ovarian cancer recurred within 6 months of chemotherapy.

Similarly, ROC curve analysis revealed that *KIF26B* expression was significantly higher in ovarian cancer chemotherapy-resistant tissues than in sensitive tissues across all drug groups, including the overall cohort, platinum-based chemotherapy subgroup, and paclitaxel subgroup, as shown in Figure 3. This increase in expression was associated with chemotherapy resistance, with area under the curve (AUC) values exceeding 0.6 in all drug groups, suggesting that high *KIF26B* expression is a strong predictor of chemotherapy resistance in ovarian cancer. Furthermore, ROC analysis revealed that *KIF26B* expression varied significantly across different stages of ovarian cancer. In poorly differentiated stage III serous ovarian cancer, *KIF26B* expression was notably higher, with an AUC value of 0.65, further supporting the role of *KIF26B* as a robust predictor of chemotherapy resistance in advanced and aggressive cases. These findings emphasize the clinical utility of KIF26B in guiding therapeutic strategies and predicting treatment responses, especially for patients with chemotherapy-resistant ovarian cancer.

### 3.2. KIF26B Is Highly Expressed in Paclitaxel-Resistant Ovarian Cancer Cells and Its Knockdown Suppresses Cell Proliferation

**Figure 4:**
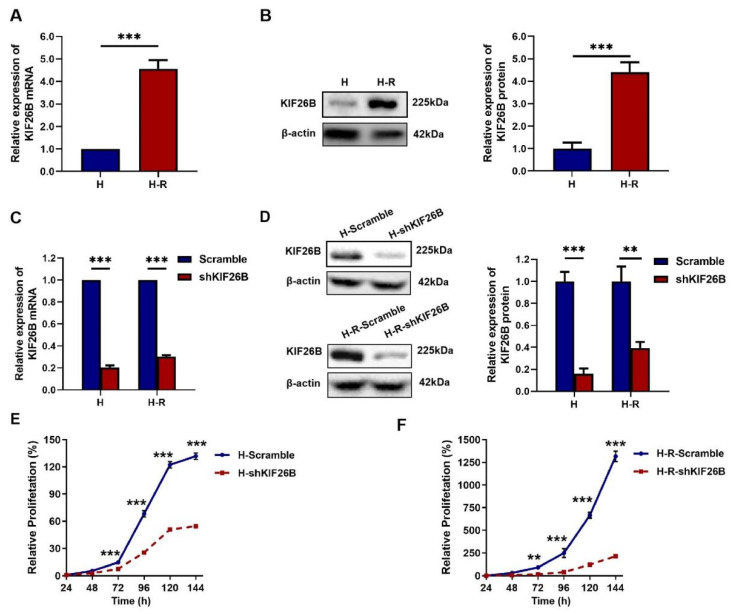
KIF26B knockdown inhibits ovarian cancer cell proliferation. (**A**) RT-qPCR analysis of *KIF26B* mRNA expression levels in H and H-R cells, *** *p* < 0.001. (**B**) Western blotting analysis of KIF26B protein expression levels in H and H-R cells, *** *p* < 0.001. H: the parent cell HeyA8; H-R: the paclitaxel-resistant ovarian cancer cell HeyA8-R. (**C**) RT-qPCR analysis of *KIF26B* mRNA expression levels in four cell lines, i.e., H-Scramble, H-shKIF26B, H-R-Scramble, and H-R-shKIF26B cells, *** *p* < 0.001. (**D**) Western blotting analysis of KIF26B protein expression levels in the same four cell lines, i.e., H-Scramble, H-shKIF26B, H-R-Scramble, and H-R-shKIF26B cells, ** *p* < 0.01, *** *p* < 0.001. (**E**,**F**) Seven-day growth curves of (**E**) H-Scramble/H-shKIF26B cells and (**F**) H-R-Scramble/H-R-shKIF26B cells. Cell proliferation was measured by the CCK-8 assay. The experiments were conducted in triplicate, and comparisons between scramble cells and shKIF26B cells treated with the same paclitaxel concentration were performed using two-way ANOVA; ** *p* < 0.01, *** *p* < 0.001.

Given the observed upregulation of KIF26B expression in ovarian cancer tissues, we next evaluated its expression in ovarian cancer cells to investigate its potential association with paclitaxel resistance. The analysis was performed on the ovarian cancer parental cells H and the corresponding paclitaxel-resistant cells H-R. The H-R cells exhibited a paclitaxel RI of 5.42 ± 0.55, calculated as the IC_50_ ratio relative to the parental H cells. RT-qPCR and Western blotting analyses confirmed that KIF26B was expressed in both lines, with its expression levels significantly higher in the paclitaxel-resistant H-R cells compared to the parental H cells (*p* < 0.001; Figure 4A,B). To investigate the functional role of KIF26B, lentiviral-mediated shRNA was used to stably knockdown KIF26B expression in both H and H-R cells. The knockdown efficiency was assessed by RT-qPCR and Western blotting, which revealed a marked reduction in both *KIF26B* mRNA and KIF26B protein levels (Figure 4C,D). Cell growth curve assays were then performed to assess the effect of KIF26B knockdown on proliferation. As shown in Figure 4E,F, compared with the scramble cells, KIF26B knockdown significantly inhibited the proliferation of both H and H-R cells, which exhibited reduced growth rates.

### 3.3. KIF26B Knockdown Enhances Paclitaxel Sensitivity in Ovarian Cancer Cells by Promoting Microtubule Polymerization

**Figure 5:**
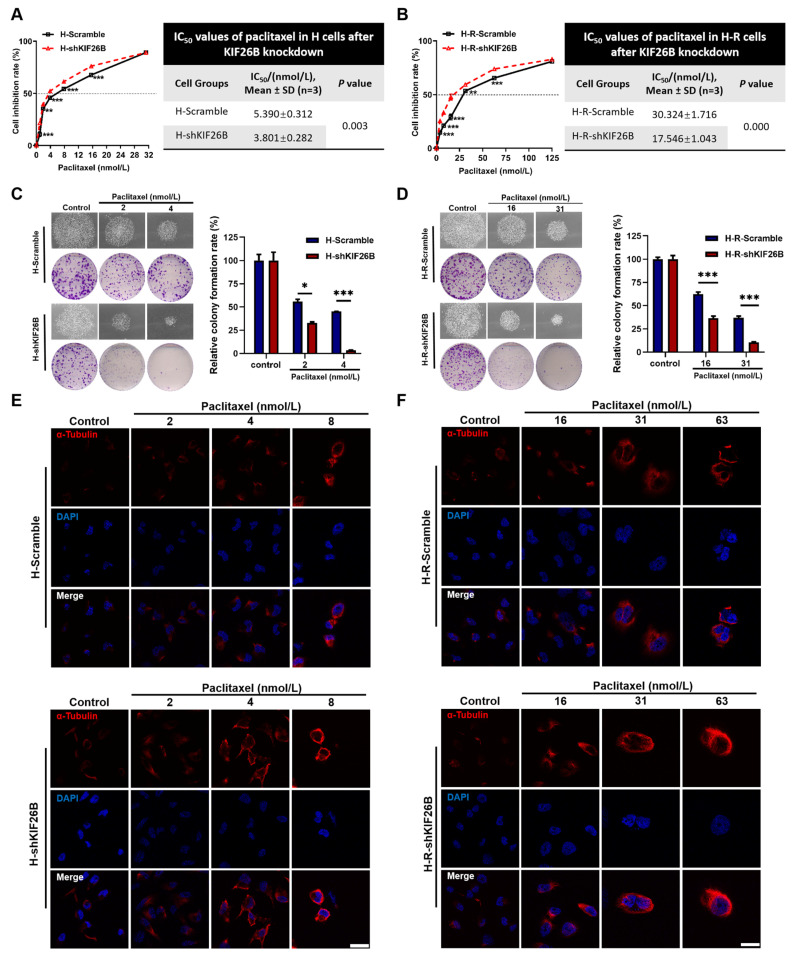
Effects of KIF26B knockdown on paclitaxel resistance in ovarian cancer cells. (**A**,**B**) Dose-dependent inhibition curves of (**A**) H-Scramble/H-shKIF26B cells and (**B**) H-R-Scramble/H-R-shKIF26B cells after 72 h of treatment with increasing concentrations of paclitaxel. In both panels, the black curve represents the Scramble control group, and the red curve represents the shKIF26B group. The IC_50_ values of paclitaxel in these two transfected cell lines are compared, ** *p* < 0.01, *** *p* < 0.001. (**C**,**D**) Scanned images of crystal violet-stained cells from the colony formation assay after 9 days of treatment with varying doses of paclitaxel in (**C**) H-Scramble/H-shKIF26B cells and (**D**) H-R-Scramble/H-R-shKIF26B cells. Relative colony formation efficiency was quantified using ImageJ software on the basis of the colony formation results, * *p* < 0.05, *** *p* < 0.001. (**E**,**F**) Representative confocal images of tubulin immunofluorescence staining following 72 h of paclitaxel treatment in (**E**) H-Scramble/H-shKIF26B cells and (**F**) H-R-Scramble and H-R-shKIF26B cells. Red fluorescence indicates α-tubulin. DAPI was used to stain nuclei. The “Merge” image shows the combined staining of α-tubulin and DAPI.

Cell viability assays were performed to determine whether KIF26B knockdown affected paclitaxel resistance in ovarian cancer cells. As shown in Figure 5A,B, KIF26B knockdown significantly reduced the survival rates of both H and H-R cells compared with those of control cells after paclitaxel treatment, indicating increased drug sensitivity. To validate these observations, colony formation assays were conducted. Consistent with the viability assay results, compared with control cells, KIF26B knockdown cells formed markedly fewer colonies in the presence of paclitaxel (Figure 5C,D), confirming that KIF26B knockdown impaired the clonogenic potential of both H and H-R cells under paclitaxel treatment.

To explore the mechanism underlying this enhanced drug sensitivity, microtubule polymerization was assessed by α-tubulin immunofluorescence staining. H-Scramble and H-shKIF26B cells were treated with 2, 4, and 8 nmol/L paclitaxel, while H-R-Scramble and H-R-shKIF26B cells were treated with 16, 31, and 63 nmol/L paclitaxel for 72 h. Red fluorescence intensity was used as a readout of microtubule formation. As shown in Figure 5E, compared with H-Scramble cells, H-shKIF26B cells displayed stronger microtubule aggregation and higher fluorescence intensity under identical paclitaxel conditions, suggesting greater susceptibility to paclitaxel-induced microtubule stabilization. Similarly, in H-R cells, compared with the Scramble cells, KIF26B knockdown further increased the fluorescence intensity under identical paclitaxel conditions (Figure 5F). Taken together, these results demonstrate that KIF26B knockdown enhanced the sensitivity of both parental and paclitaxel-resistant ovarian cancer cells to paclitaxel by promoting microtubule polymerization.

### 3.4. KIF26B Knockdown Induces Cell Cycle Arrest and Apoptosis in Ovarian Cancer Cells

**Figure 6:**
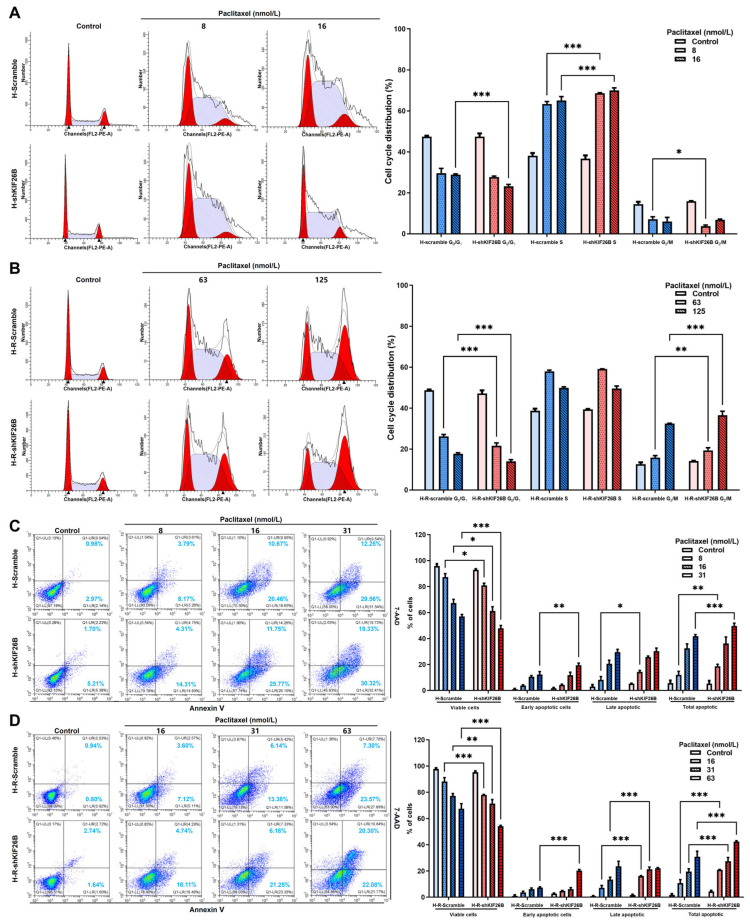
Effects of KIF26B knockdown on cell cycle distribution and apoptosis in response to paclitaxel treatment. (**A**,**B**) Cell cycle distribution analysed by flow cytometry and ModFit after 72 h of treatment with increasing concentrations of paclitaxel in (**A**) H-Scramble/H-shKIF26B cells and (**B**) H-R-Scramble/H-R-shKIF26B cells. In the flow cytometry histograms, red regions correspond to the populations of cells in the G_0_/G_1_ phase (left peak, 2N DNA content) and the G_2_/M phase (right peak, 4N DNA content); purple regions represent cells in the S phase (DNA content between 2N and 4N). The overlaid black line represents the fitted curve. Quantitative analysis of the percentage of cells in the G_0_/G_1_, S, and G_2_/M phases after exposure to varying paclitaxel concentrations for 72 h. (**C**,**D**) Representative flow cytometry plots of apoptotic (**C**) H-Scramble/H-shKIF26B cells and (**D**) H-R-Scramble/H-R-shKIF26B cells after 72 h of paclitaxel exposure, as assessed by PE Annexin V/7-AAD double staining; quantification of apoptosis rates, including viable, early apoptotic, late apoptotic, and total apoptotic populations. All experiments were repeated three times, and statistical comparisons between scramble cells and shKIF26B cells at the same paclitaxel concentration were performed using two-way ANOVA. * *p* < 0.05, ** *p* < 0.01, and *** *p* < 0.001.

To further elucidate the cellular mechanisms underlying the increased paclitaxel sensitivity induced by KIF26B knockdown, flow cytometry was used to analyse cell cycle progression and apoptosis in both the H and H-R cell lines. The results revealed that following KIF26B knockdown, paclitaxel treatment significantly increased S-phase arrest in H cells (Figure 6A) whereas G_2_/M-phase arrest was markedly enhanced in H-R cells (Figure 6B); these findings indicate a strong inhibitory effect on the cell cycle, with statistically significant differences (*p* < 0.05). Additionally, KIF26B knockdown significantly increased the rate of paclitaxel-induced apoptosis, especially in H-R cells. As the paclitaxel concentration increased, the proportion of live cells in both the control and KIF26B knockdown groups decreased in a concentration-dependent manner. Moreover, early apoptosis, late apoptosis, and total apoptotic cell proportions were consistently greater in the KIF26B knockdown group than in the control group (Figure 6C,D). These results suggest that KIF26B knockdown enhances paclitaxel-induced apoptosis and cell death in ovarian cancer cells, indicating that KIF26B may play a critical role in regulating the resistance of ovarian cancer cells to paclitaxel.

### 3.5. SLC7A11 Is Functionally Associated with KIF26B in the Paclitaxel Response of Ovarian Cancer

**Figure 7:**
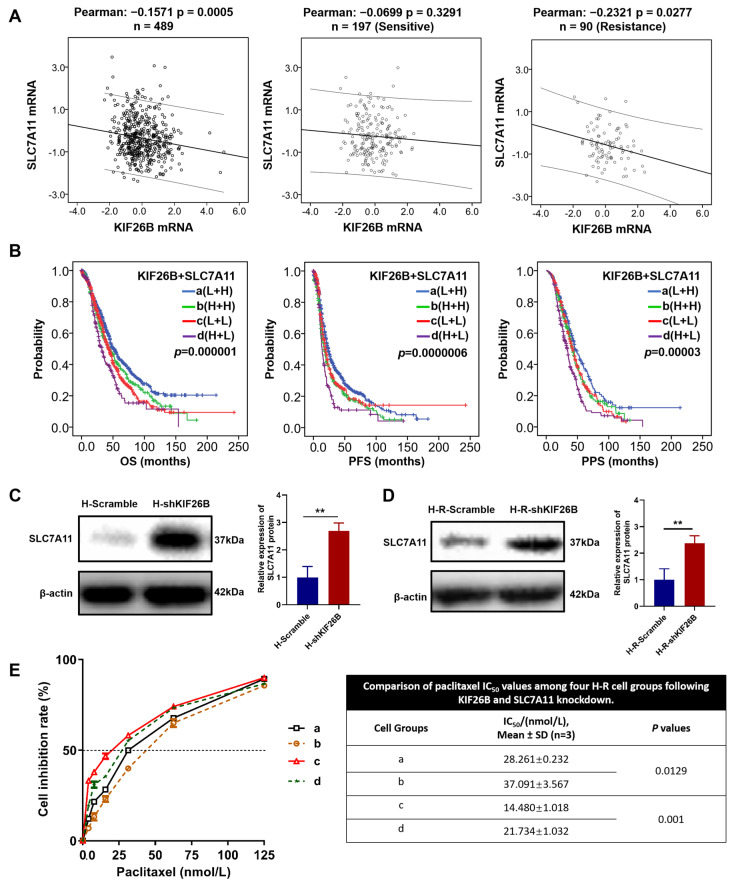
Expression patterns of KIF26B and SLC7A11 in ovarian cancer and their associations with prognosis and paclitaxel response. (**A**) Analysis of the correlation between *KIF26B* and *SLC7A11* expression in 489 ovarian cancer tissues derived from TCGA transcriptome data. (**B**) Combined analysis of KIF26B and SLC7A11 expression in ovarian cancer patients revealed significant differences in OS, PFS and PPS between the combined expression groups: a (L + H), b (H + H), c (L + L), and d (H + L). OS, overall survival; PFS, progression-free survival; PPS, post-progression survival. (**C**,**D**) Western blotting analysis of SLC7A11 protein levels in (**C**) H-Scramble/H-shKIF26B cells and (**D**) H-R-Scramble/H-R-shKIF26B cells. Data were normalized to β-actin and are presented as mean ± SD of three independent experiments, ** *p* < 0.01. (**E**) Dose-dependent inhibition curves and IC_50_ values of paclitaxel in H-R cells with combinatorial knockdown of KIF26B and SLC7A11. Cells with stable KIF26B knockdown or control (Scramble) stable lines were transfected with siRNA targeting SLC7A11 or a non-targeting control (si-NC). Groups a-d were then treated with a paclitaxel gradient for 72 h. Cell inhibition rate was quantified using the CellTiter-Glo luminescent assay. Data points (mean ± SD of three independent experiments) represent the dose-dependent inhibition, and the corresponding IC_50_ are displayed within the panel. Group definitions: a, H-R-Scramble + si-NC; b, H-R-Scramble + si-SLC7A11 A; c, H-R-shKIF26B + si-NC; d, H-R-shKIF26B + si-SLC7A11.

To further explore the downstream mechanisms underlying the effects of KIF26B knockdown, the relationship between KIF26B and SLC7A11 was examined at both the transcriptome and protein levels in ovarian cancer. Using TCGA transcriptome data from 489 ovarian cancer tissues, a significant negative correlation between *KIF26B* and *SLC7A11* expression was observed in the total cohort as well as in 90 chemoresistant tissues (Figure 7A). No correlation was detected in 197 chemosensitive tissues, suggesting that the negative association between the two genes may be enriched in chemoresistant ovarian cancer. Our previous research revealed that low SLC7A11 expression is significantly associated with poor OS, PFS, and PPS [11]. Given the potential inhibitory relationship between KIF26B and SLC7A11 in ovarian cancer, we explored the combined effect of these two genes on prognosis. Kaplan–Meier survival analysis of 1815 patients stratified into four groups according to combined KIF26B and SLC7A11 expression revealed distinct survival outcomes (Figure 7B). Patients with high KIF26B expression and low SLC7A11 expression (group d, H + L) had the poorest prognosis, whereas patients with low KIF26B expression and high SLC7A11 expression (group a, L + H) had the most favourable outcomes. Western blotting confirmed the negative regulatory relationship at the protein level. The expression of KIF26B was significantly increased upon KIF26B knockdown within both H cells and H-R cells (Figure 7C,D). The functional relevance of this association was investigated by transiently silencing SLC7A11 in KIF26B-depleted H-R cells, followed by assessment of paclitaxel sensitivity. As shown in Figure 7E, compared with KIF26B knockdown alone, the combined knockdown of SLC7A11 and KIF26B partially reversed this effect, leading to a higher IC_50_. These findings indicate that silencing SLC7A11 attenuates the enhanced paclitaxel sensitivity induced by KIF26B knockdown, suggesting a functional association between SLC7A11 and KIF26B in regulating the paclitaxel response.

**Figure 8:**
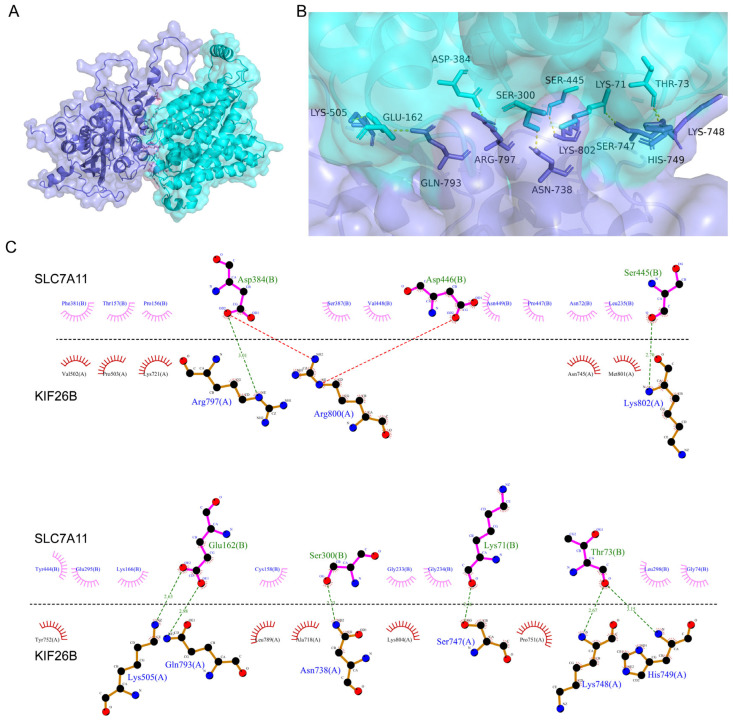
Protein-protein docking analysis of the interaction between KIF26B and SLC7A11. (**A**) Predicted three-dimensional docking model of the KIF26B-SLC7A11 complex generated using the HDOCK server. KIF26B is shown in purple, and SLC7A11 is shown in cyan. The putative interaction interface is highlighted. (**B**) Enlarged view of the predicted interaction interface, highlighting residues from KIF26B-SLC7A11 that are implicated in intermolecular contacts. Putative hydrogen bonds and salt bridge interactions are indicated by dashed lines. (**C**) Two-dimensional schematic representation of the predicted intermolecular interactions between KIF26B-SLC7A11 generated using LigPlot+ version 2.2.4. Putative hydrogen bonds and salt bridge interactions are indicated by dashed lines, while hydrophobic contacts are shown as red arcs. Residues at the predicted interaction interface are labeled.

Based on this correlation between KIF26B and SLC7A11, protein–protein docking was employed to computationally evaluate their potential direct physical interaction. Among the predicted docking models generated by the HDOCK server, the top-ranked model (model_1) was selected for subsequent analysis. Interfacial residues involved in hydrogen bonding, salt bridge formation, and hydrophobic interactions were identified using LigPlot+ version 2.2.4, and the predicted protein–protein docking conformation was visualized using PyMol version 2.2.0. As shown in Figure 8, multiple residues were predicted to participate in hydrogen bond interactions between KIF26B and SLC7A11, including Arg-797 of KIF26B and Asp-384 of SLC7A11. In addition, a salt bridge was observed between Arg-800 of KIF26B and Asp-384 of SLC7A11, while hydrophobic interactions were detected between Val-502 of KIF26B and Phe-381 of SLC7A11. Based on these intermolecular interactions, the KIF26B and SLC7A11 docking model achieved a top docking score of −6904.19, suggesting a favorable predicted interaction. Collectively, these results provide structural support for a potential direct association between KIF26B and SLC7A11.

## 4. Discussion

Ovarian cancer holds a prominent position among malignancies of the female reproductive system and is among the most lethal cancers affecting women. Ovarian cancer treatment typically involves surgery, chemotherapy, and targeted therapies. However, owing to tumour heterogeneity and the development of resistance to treatment, many patients experience relapse after initial therapy. Understanding the molecular mechanisms underlying ovarian cancer is crucial for the development of more precise therapeutic strategies, which can help reduce recurrence rates and improve treatment outcomes. Furthermore, exploring new drug therapeutic targets is essential for enhancing treatment success and extending patient survival.

Kinesin family proteins (KIFs) are a group of motor proteins widely present in eukaryotes that play crucial roles in physiological processes such as embryonic development, axonal transport, and cell division. Specifically, KIFs are key players in processes such as spindle assembly, chromosome segregation, and cytokinesis. Since mitosis is a validated target for anticancer therapies, KIFs are considered promising therapeutic targets for cancer biotherapy [12]. In recent years, increasing evidence has shown that kinesins play critical roles in the initiation and progression of various cancers. For instance, studies reported that the expression of KIF4A, a member of the kinesin-4 family, is upregulated by at least fivefold in most lung cancer patients and its overexpression is strongly associated with poor prognosis [13]. Additionally, KIF11 and KIF14 are significantly associated with gastrointestinal cancers [14]. Furthermore, other studies using mass spectrometry, immunoprecipitation, and pull-down assays have revealed that KIF11 interacts with the tumour necrosis factors TRAF4 and DR6, contributing to tumorigenesis in ovarian malignancies [15],and Kaplan -Meier, Oncomine, and gene expression profile analyses further suggest that KIF11 overexpression is correlated with poor prognosis in epithelial ovarian cancer patients [16]. Given the critical role of KIFs during mitosis, KIF family members have garnered considerable attention in the search for novel mitotic drug targets [17]. Accumulating evidence has demonstrated that multiple KIFs are closely involved in the development of chemotherapy resistance in various cancers, specifically, KIFC3 and MCAK, have been implicated in the development of chemotherapy resistance [18,19]. Additionally, KIF20B and KIF4A, have also been shown to be involved in the regulation of tumor chemotherapy resistance [20,21]; inhibition of KIF20B can sensitize hepatocellular carcinoma cells to microtubule-targeting agents by blocking cytokinesis, while KIF4A participates in the formation of chemotherapy resistance in lung cancer by regulating the intracellular trafficking of lung resistance-related protein. KIF20A, another member of the kinesin family that is involved in vesicular transport and cell division [22]; it is highly expressed in ovarian clear cell carcinoma [23] and is closely associated with recurrence and platinum drug resistance [24]. Collectively, these studies suggest that the KIF family not only maintains basic cellular life activities but also serves as important regulatory nodes in tumor drug resistance.

Among KIF family members, KIF26B has been linked to the occurrence and progression of multiple cancers, including breast cancer [25], colorectal cancer [26], hepatocellular carcinoma [27], NSCLC [9], and gastric cancer [28]. In these human cancers, upregulated KIF26B expression is consistently associated with malignant clinical-pathological features, such as increased tumour size, high tumour grade, lymph node metastasis, and distant metastasis, all of which contribute to poor prognosis [8,26,28]. However, the expression profile, prognostic value, and functional role of KIF26B in ovarian cancer remain unclear. On the basis of these findings, in this study, a systematic analysis of the relationship between KIF26B and ovarian cancer was conducted using bioinformatics through open-access databases. The results revealed that both KIF26B mRNA and protein expression levels were significantly elevated in ovarian cancer. Further analysis demonstrated that KIF26B expression is significantly upregulated in platinum and taxane-resistant ovarian cancer tissues, indicating that high KIF26B expression in resistant tissues can serve as an effective predictor of chemotherapy resistance to platinum and taxane. Consistent with these bioinformatics findings, we verified that KIF26B expression was significantly increased in paclitaxel-resistant H-R ovarian cancer cells compared with parental H cells. This phenomenon suggests that the high expression of KIF26B may be closely associated with paclitaxel resistance in ovarian cancer cells. To validate this hypothesis, we transfected ovarian cancer cells H and paclitaxel-resistant H-R cells with lentivirus-mediated KIF26B shRNA to interfere with KIF26B expression. Functional experiments demonstrated that downregulation of KIF26B significantly inhibited the proliferation and colony formation ability in both ovarian cancer cells H and paclitaxel-resistant H-R cells. Meanwhile, KIF26B knockdown was confirmed to promote microtubule polymerization, thereby inducing cell cycle arrest, promoting apoptosis, and ultimately reducing the resistance of ovarian cancer cells H and paclitaxel-resistant H-R cells to paclitaxel. The above experimental data not only verified the initially proposed hypothesis but also were consistent with relevant research findings: previous studies have shown that KIF26B can promote malignant tumor progression in gastric cancer and medulloblastoma by activating different signaling pathways, respectively [28,29]. Furthermore, previous studies have confirmed that long non-coding RNA AC105118.1 can promote oxaliplatin resistance in colorectal cancer cells by regulating the miR-378a-3p/KIF26B axis [30]. This study directly focuses on KIF26B-mediated regulation of tumor drug resistance, which provides theoretical support for the reliability of KIF26B as a therapeutic target related to tumor drug resistance and indirectly validates the findings of this study regarding the role of KIF26B in ovarian cancer paclitaxel resistance.

We further explored the molecular mechanism by which KIF26B regulates paclitaxel resistance in ovarian cancer. Previous studies have shown that in malignant tumors such as gastric cancer and liver cancer, KIF26B can promote tumor cell proliferation and metastasis by activating the PIK3CA/AKT gene and its related pathway [27,28,29]. Meanwhile, PIK3CA can regulate tumor cell nutrient uptake by inhibiting SLC7A11 expression, suggesting a potential negative regulatory relationship between KIF26B and SLC7A11 in tumor tissues [31]. As a key amino acid transporter, SLC7A11 is involved in glutamine uptake and intracellular glutathione synthesis, which can enhance cellular antioxidant capacity and protect tumor cells from oxidative stress damage induced by chemotherapeutic drugs [32]. Therefore, we conducted a correlation analysis of KIF26B and SLC7A11 expression. The results revealed no correlation between the two genes in sensitive tissues but a potential negative correlation in drug-resistant tissues. Further combined prognostic analysis demonstrated that the coexpression levels of KIF26B and SLC7A11 are closely related to patient prognosis of ovarian cancer. Specifically, patients with high KIF26B expression and low SLC7A11 expression had significantly poorer survival rates. These findings indicate that KIF26B and SLC7A11 may jointly contribute to ovarian cancer progression and drug resistance regulation, thereby affecting patient outcomes. Similarly, SLC7A11 plays a critical role in the oxidative stress response and survival of tumor cells, and its expression level has been confirmed to be associated with poor prognosis in several cancers [33,34], as well as with poor prognosis and paclitaxel resistance in ovarian cancer [11,35]. To further clarify the regulatory association between KIF26B and SLC7A11 in paclitaxel resistance of ovarian cancer, we conducted functional analyses, and the results further implicated SLC7A11 in the KIF26B-mediated cellular response to paclitaxel in ovarian cancer cells. The results of molecular docking experiments showed that the top docking score of the KIF26B and SLC7A11 docking model was −6904.19, suggesting a favorable predicted direct interaction between the two. Collectively, these results provide structural support for a potential direct association between KIF26B and SLC7A11. Nonetheless, on the basis of our findings, further exploration is needed to elucidate the specific roles of KIF26B and SLC7A11 in ovarian cancer.

Taken together, our work has significant clinical implications, as paclitaxel is among the frontline drugs for ovarian cancer and paclitaxel resistance is a major challenge in treatment efficacy. KIF26B knockdown may not only restore paclitaxel efficacy but also offer a new therapeutic target for patients with drug-resistant ovarian cancer. However, this study has certain limitations. While this study provides preliminary evidence for the interaction between KIF26B and SLC7A11 in paclitaxel resistance, the specific molecular mechanisms underlying this interaction remain unclear and require further investigation. In particular, targeted experimental validation should be performed to verify the potential physical association KIF26B and SLC7A11 proteins, which is essential for corroborating the interaction tendency predicted by bioinformatic docking analysis. Future studies should focus on validating these findings in vivo, exploring the roles of KIF26B and SLC7A11 in the tumour microenvironment, and investigating their potential involvement in resistance to other chemotherapeutic agents. Additionally, clinical sample validation of KIF26B and SLC7A11 regulation is needed to confirm and expand upon our findings. Furthermore, incorporating more clinical data will help clarify the role of KIF26B and SLC7A11 in paclitaxel resistance, advancing the development of personalized treatment strategies for ovarian cancer.

## 5. Conclusions

(1) Bioinformatics analyses revealed that KIF26B is highly expressed in ovarian cancer tissues and is associated with paclitaxel resistance and poor prognosis. Paclitaxel resistance was decreased by KIF26B knockdown in ovarian cancer cells, as demonstrated by reduced proliferation and clonogenicity, enhanced tubulin polymerization capacity, G_0_/G_1_-phase cell-cycle arrest, and increased apoptosis, supporting KIF26B as a potential therapeutic target.

(2) A potential mechanism was suggested that KIF26B may contribute to paclitaxel resistance by modulating SLC7A11 expression. In ovarian cancer, KIF26B and SLC7A11 showed an inverse association, and their combined expression exhibited greater prognostic value than either gene alone. Functional analyses further implicated SLC7A11 in the KIF26B mediated cellular response to paclitaxel.

## Data Availability

The original contributions presented in the study are included in the article; further inquiries can be directed to the corresponding author.
